# Accelerated crystallization of colloidal glass by mechanical oscillation

**DOI:** 10.1038/s41598-017-01484-y

**Published:** 2017-05-02

**Authors:** Nobutomo Nakamura, Kyosuke Inayama, Tasuku Okuno, Hirotsugu Ogi, Masahiko Hirao

**Affiliations:** Graduate School of Engineering Science, Osaka University, Toyonaka, Osaka 560-8531 Japan

## Abstract

Crystallization of a hard-sphere colloidal glass by mechanical oscillation is investigated, and accelerated crystallization is found at a specific frequency. The crystallization frequency increases as attractive force between particles increases, indicating that interparticle interaction affects the crystallization frequency. Time scale of the mechanical oscillation is different from that of the slow relaxation, and notable relationship with the low-frequency mode is not observed. The experimental results are not explained by the previously proposed model for crystallization by oscillatory shear. Conversely, we speculate that activations of the fast relaxation and particle motion in crystal-like clusters are possible causes of the observations.

## Introduction

In glass-forming materials, there are characteristic vibrational behaviors, slow (*α*) relaxation, fast (*β*) relaxation, and Boson peak, and their relationships with structure and dynamics have been investigated^1–5^. Among the studies, it is empirically found that ultrasonic annealing crystallizes Pd-based metallic glass below the glass transition temperature^6^. The result is explained by the view that atomic motions associated with relaxation are stochastically resonant with the motions caused by ultrasonic vibrations. Contribution of relaxation to crystallization is indicated also in polymers^7, 8^. These studies imply that there are vibrational modes that are related with structural evolutions in amorphous solids. In amorphous solids, crystallization at elevated temperatures is used for fabricating nanocrystalline materials^9–11^. If activating a specific mode accelerates a structural evolution, stress-wave agitation can be an alternative way to control crystallization of amorphous solids. Purpose of the present study is understanding relationship between the stress-wave agitation and the crystallization of amorphous solids.

To investigate the relationship, we focus on an acceleration phenomenon of crystallization in a colloidal glass. Frequency of phonon modes ranges widely in actual materials, exceeding THz order. Excitation of a single mode among them is difficult. For solving the problem, we use a colloidal system. Since the finding that a hard-sphere colloid shows phases similar to those of atomic systems^12^, colloids are studied for simulating structural evolutions in atomic systems^13–16^. Size of colloidal particles is larger and relaxation time is longer than those of atomic materials, which allows the dynamic particle-scale analysis inside a colloidal system that cannot be performed in atomic materials. In colloidal glasses, the low-frequency mode^17–19^, analogous to the Boson peak, is observed. Particle motion in cages and a jump to a different cage are also observed^14, 20^. Thus, colloidal systems can be model materials for studying dynamic properties of atomic materials.

Effect of oscillatory shear on structure of colloidal systems has been evaluated^21–23^, and phonon modes are also investigated^24^. Smith *et al*.^23^ investigated the crystallization by shear oscillations at three different frequencies (1, 10, and 70 Hz) in colloidal gels and found that oscillation with the highest frequency crystallizes colloidal gels with smaller amplitude. In the study, a model is proposed for explaining the results; when a time scale, a function of frequency and amplitude, becomes smaller than a specific value, crystallization occurs. The model indicates that above a specific frequency, crystallization occurs, and presence of a phonon mode around which crystallization is accelerated is not indicated. However, in the present study, we investigate frequency dependence of crystallization in more detail using a hard-sphere colloidal system, and it is revealed that there is a specific frequency around which crystallization is significantly accelerated, which is not explained by the previously proposed model.

## Results and Discussion

A colloidal glass is formed by settling silica particles dispersed in a solution on a bottom surface of a sample cell by centrifugation. Mechanical oscillation is applied to the colloidal glass by moving the oscillator inserted into the colloidal suspension. The oscillator is not touching the colloidal glass. Structure of the colloidal glass is observed by using a confocal laser scanning microscope. Details of the sample and experimental setup are described in Methods. The oscillator is the square rod, and tip of the oscillator is flat. In the following experiments, crystallization below the tip is evaluated. When the oscillator is moved horizontally in the suspension, a shear wave that propagates in the thickness direction of the colloidal glass is excited from the flat face at the tip. The shear wave propagates with damping, and the amplitude decreases exponentially, exp (−*z*/*δ*), with increasing distance from the tip, *z* ^25^. The decay length, *δ*, is given by *δ* = (2*η*_*L*_/*ωρ*_*L*_)^1/2^, where *η*_*L*_ is the viscosity of the liquid, *ρ*_*L*_ the density of the liquid, and *ω* the angular frequency of the oscillator. In the present experimental condition, *δ* is 191 *μ*m at 30 Hz and 105 *μ*m at 100 Hz (see Supplementary Fig. S1). These values are larger than the gap, ~10 μm, between the tip of the oscillator and the top surface of the colloidal glass. Therefore, shear deformation is caused in the colloidal glass.

**Figure 1:**
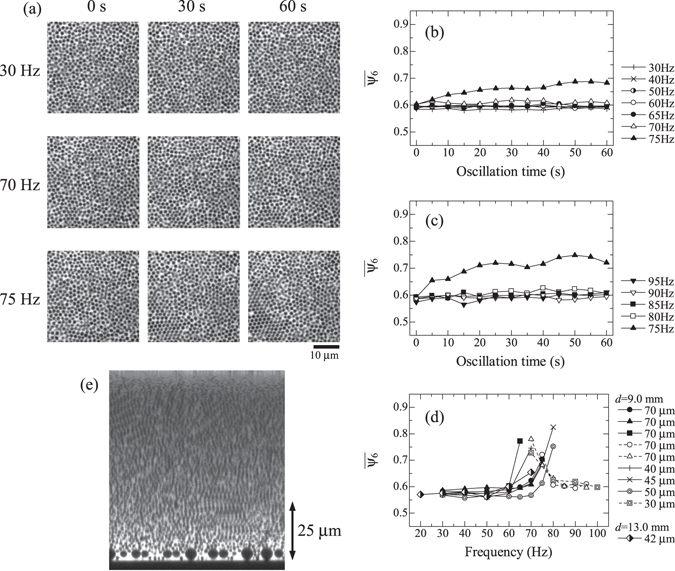
Crystallization of colloidal glasses under mechanical oscillations. (**a**) Microscopy images taken before and after 30 and 60 s oscillations are shown. They are parts of original images. (**b**,**c**) Evolution of the averaged bond orientational order parameter with increasing and decreasing the oscillation frequency. (**d**) Averaged bond orientational order parameter after oscillations for 60 s. (**e**) A cross-sectional image taken after 60-s oscillation at 75 Hz. The arrow indicates a typical height where 2D images are taken for analysis.

First, we evaluate effect of mechanical oscillations at 30–100 Hz on structural evolution of colloidal glasses. Just after a sample is prepared, it is set on the microscope stage, and mechanical oscillation is applied. Amplitude of the oscillation is kept to be 5 *μ*m. After oscillation is applied at a frequency for 5 s, a 2D image inside a colloidal glass is taken. Then, 5-s oscillation is applied again at the same frequency, followed by taking an image. The sequence is repeated twelve times at the frequency until total oscillation duration becomes 60 s. Then, oscillation frequency is changed, and the same procedure is repeated. Figure 1(a) shows representative microscope images when the oscillation frequency is increased from 30 to 75 Hz. The original image size is 71.7 *μ*m by 71.7 *μ*m. At 70 Hz and lower frequencies, notable structural evolution is not observed. However, crystalline regions are clearly formed at 75 Hz.

To quantify the degree of the crystallization, 2D local bond orientational order parameter, ${\psi }_{6i}=|\frac{{\sum }_{m=1}^{{n}_{i}}{e}^{j6{\theta }_{mi}}}{{n}_{i}}|$ ^19^, is calculated. Here, *n*_*i*_ is the number of the nearest neighbors around particle *i*, and *θ*_*mi*_ is the angle between the *x* direction, the horizontal direction in an image, and the vector from particle *i* to a neighboring particle *m*. Neighboring particles within 2 *μ*m from particle *i*, located closer than the first minimum in the radial distribution function, are used in the calculation. When the neighboring particles show six-fold symmetry, *ψ*_6*i*_ becomes unity, and it becomes smaller as the structure becomes random. Figure 1(b) plots the evolutions of an average value of *ψ*_6*i*_, $\overline{{\psi }_{6}}=\frac{\sum _{i=1}^{{n}_{I}}{\psi }_{6i}}{{n}_{I}}$, over particles (1, …, *n*_*I*_) in each image, where *n*_*I*_ is the number of particles in an image. At 30–70 Hz, $\overline{{\psi }_{6}}$ is almost unchanged with oscillation time. In contrast, it increases at 75 Hz with time. Evolutions of $\overline{{\psi }_{6}}$ with decreasing the oscillation frequency measured for a different sample are plotted in Fig. 1(c). Crystallization is not observed at higher frequencies, but it clearly occurs at 75 Hz. Figure 1(d) plots the $\overline{{\psi }_{6}}$ measured after 60-s oscillation at each frequency. Results of a few independent measurements are plotted together. A peak frequency where crystallization progresses appears around 70 Hz, indicating presence of a specific frequency that accelerates the crystallization of colloidal glasses. Figure 1(e) shows a cross-sectional image taken after crystallization is caused by 75-Hz oscillation.

**Figure 2:**
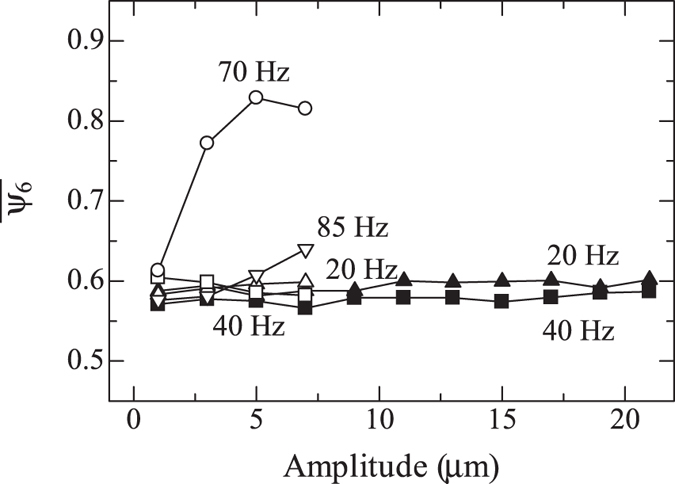
Averaged bond orientational order parameters after 30-s oscillations with increasing amplitude at different frequencies. Open symbols represent the experimental results when oscillations are applied to a sample with changing the frequency in the order of 20, 40, 85, and 70 Hz; the order is 1 *μ*m at 20 Hz, 3 *μ*m at 20 Hz, …, 7 *μ*m at 20 Hz, 1 *μ*m at 40 Hz, …, and 7 *μ*m at 70 Hz. Filled symbols represent the experimental results for different samples.

High crystallization efficiency around 75 Hz is clearly observed also in amplitude dependence of crystallization. A sample is prepared, and crystallization is monitored with increasing the amplitude at different frequencies. At first, 20-Hz oscillation with 1-*μ*m amplitude is applied for 30 s, and a 2D image is taken. This sequence is repeated with increasing amplitude until 7 *μ*m. After that, the frequency is changed to 40 Hz, and oscillation amplitude is increased from 1 *μ*m to 7 *μ*m. The frequency is changed in the order of 20, 40, 85, and 70 Hz. The results are shown in Fig. 2. At 20 and 40 Hz, notable crystallization is not observed. At 85 Hz, $\overline{{\psi }_{6}}$ increases slightly at 7 *μ*m, but the remarkable crystallization occurs at 70 Hz. Two samples are also prepared, and amplitude dependence is evaluated at 20 and 40 Hz for each sample. At both frequencies, notable crystallization does not occur until the amplitude of 21 *μ*m. These results demonstrate how strongly ∼75-Hz oscillation accelerates the crystallization.

Above results indicate that there is a specific vibrational mode accelerating crystallization in a colloidal glass. We expect that the mode is associated with the phase transition from glass to crystal phases. However, there are other possible causes we have to consider. When mechanical oscillation is applied to a solid, resonance vibrations are excited at resonance frequencies. In the present colloidal glass, resonance vibrations in the thickness direction can occur. At the resonance frequencies, oscillation amplitude is amplified, and crystallization should be accelerated. Because the resonance frequencies are inversely proportional to the sample height, we can discuss its contribution by measuring the sample-height dependence of the crystallization frequency. Samples are prepared by changing the amount of the colloidal suspension poured into the sample cell. For the samples, crystallization with increasing and decreasing the oscillation frequency is monitored. The results are shown in Fig. 1(d). Peak frequency where crystallization occurs fluctuates, but it is almost independent of the sample height, indicating that resonance vibration of the sample is not the cause of the crystallization. The resonance vibration of the suspension in the sample cell, which is associated with the dimension of the sample cell, is a possible cause too. Inner diameter, *d*, of the hole where the colloidal suspension is poured is 9 mm in the present sample cell. If the crystallization frequency is associated with the vibration of the suspension, the frequency should change depending on *d*. To evaluate effect of the hole diameter on the crystallization, a sample cell with the through hole of 13-mm diameter is prepared, and the crystallization of the colloidal glass in the sample cell is monitored (microscopy images are shown in Supplementary Fig. S2). Duration of oscillation at each frequency is 10 s, and the result is shown in Fig. 1(d). The crystallization occurs around 70 Hz, and this result indicates that the hole size does not affect the crystallization frequency. Resonance vibration of the aluminium rod is also a possible cause. When the resonance happens, amplitude at the tip of the rod is amplified. In the present setup, the rod shows a cantilever shape, and the bending resonance is most likely to occur. The resonance frequency^26^ is calculated to be 740 Hz, which is far above the observed crystallization frequency. Thus, the resonance vibrations associated with the measurement setup are not affecting the present results.

**Figure 3:**
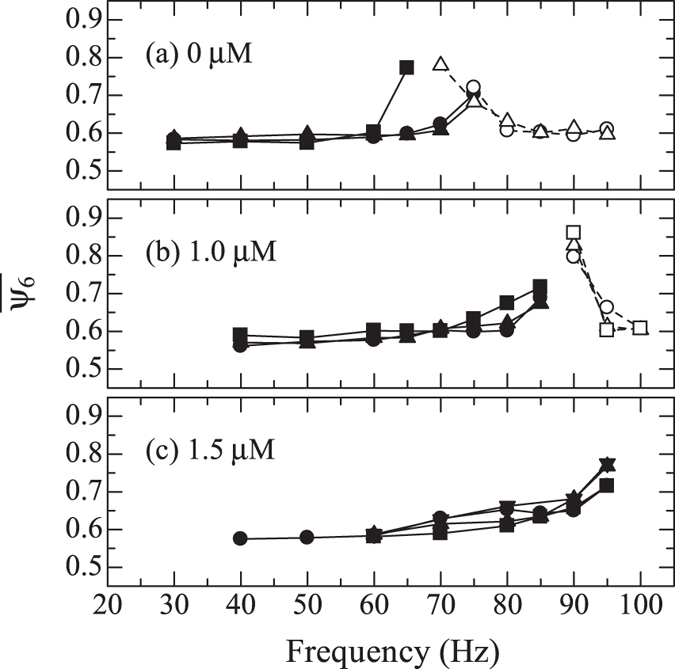
Averaged bond orientational order parameters after oscillations for 60 s. Polymer concentrations are (**a**) 0.0, (**b**) 1.0, and (**c**) 1.5 *μ*M.

To confirm that the observed crystallization frequency reflects the interaction between particles, effect of interparticle interaction on the crystallization frequency is evaluated. If the crystallization accelerated phenomenon is originating from activation of a vibrational mode associated with the particle motions, the crystallization frequency should change depending on the interaction strength. Adding polymers into a colloidal suspension is a way to modify the interaction; attractive interaction through the depletion force is given^27^. Stronger attractive interaction increases the macroscopic stiffness of a colloidal glass, and crystallization frequency will be higher. Colloidal suspensions including poly(sodium 4-styrensulfonate) (PSS) of 1.0 and 1.5 *μ*M are prepared, and colloidal glasses are formed in the same manner as the above experiments. The average molecular weight of the PSS is 70000. Evolution of $\overline{{\psi }_{6}}$ with 5-*μ*m oscillation is measured, and it is plotted in Fig. 3. The results at the corresponding sample height (70 *μ*m) for 0 *μ*M in Fig. 1(d) are plotted in Fig. 3(a). As the polymer concentration increases, the crystallization frequency moves toward higher value: 90 and 95–100 Hz at 1.0 and 1.5 *μ*M, respectively. These results support the view that the crystallization frequency reflects the interaction between colloidal particles.

One may consider that oscillation history affects the crystallization frequency, because in the above experiments oscillation frequency is increased or decreased gradually and rejuvenating is not performed before each oscillation. However, the effect should be negligibly small. 20 and 40 Hz oscillations (filled symbols) in Fig. 2 and 95 Hz oscillation in Fig. 1(c) are applied to samples without applying oscillations at different frequencies in advance. In the samples, crystallization is not caused by mechanical oscillations. We have applied mechanical oscillations at around 75 Hz to samples without applying oscillations in advance. Then, crystallization occurred immediately. These results confirm that the oscillation history hardly affects the crystallization. In Fig. 1(d), crystallization frequency fluctuates, and an average value and standard deviation are 73.6 Hz and ±5 Hz (±6.8%), respectively. We estimate effect of the volume-fraction fluctuation on the crystallization frequency by calculating a bulk modulus of a hard sphere system. In the estimation, it is assumed that the crystallization frequency *f* is proportional to a square root of the bulk modulus *K*, *f* ∝ *K* ^1/2^. *K* is calculated from *K* = −*Vdp*/*dV*, where *p* = *Z*(*V*)*RT*/*V*. *p*, *R*, *T*, and *V* are the pressure, gas constant, temperature, and molar volume of the system, respectively. *Z*(*V*) is the compressibility factor, for which we use the reported equation for a solid state^28^. According to the above calculation, ±6.8% fluctuation in the frequency corresponds to ±0.01 change in the volume fraction around 0.59, which is a conceivable fluctuation.

In the experimental results reported by Smith *et al*.^23^, crystallization occurs when the frequency exceeds a specific frequency, and it is explained by considering a model that involves the shear-assisted escape of a particle from the attractive potential. Shear strain pulls each particle away from its neighbors, and the probability for escape from the interparticles attraction increases by applying oscillatory shears. They propose that when the escape time, a function of the frequency and strain amplitude, drops below a characteristic time, crystallization occurs. It successfully explains the crystallization observed in the previous study, but this model cannot explain the present results.

**Figure 4:**
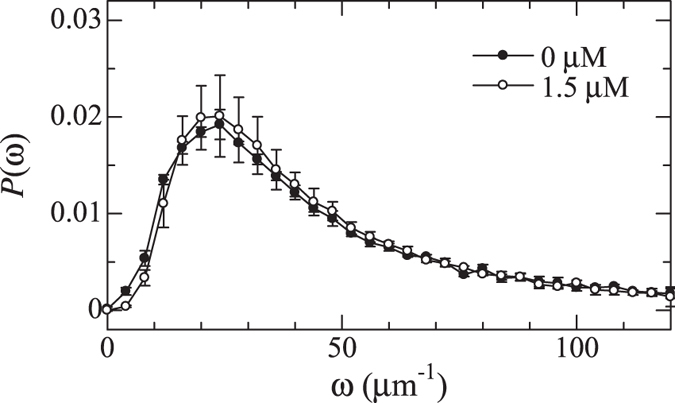
Density of states of colloidal glasses at polymer concentrations of 0.0 and 1.5 *μ*M.

We here consider the relationship between the crystallization frequency and time scales of particle motions. As described in the introduction, there are particle motions with different time scales in colloidal systems. One is the slow relaxation observed near the glass transition^14^. The time scale diverges as the glass transition is approached, and it should be significantly larger than the time scale of the applied mechanical oscillation. Therefore, the slow relaxation cannot be a cause of the present result. Another time scale is associated with the low-frequency mode, analogous to the Boson peak. In atomic materials, its time scale is smaller than that of the slow relaxation. Vibrational density of states (DOS) of condensed colloidal systems is calculated recently, and presence of the low-frequency mode is confirmed like atomic glasses^17–19^. We calculate the DOS using the normal mode analysis referring to the studies, and contribution of the low-frequency mode to the crystallization is considered. Using particle displacements from the time-average position (*u*_*i*_(*t*), *v*_*i*_(*t*)) = (*x*_*i*_(*t*) − 〈*x*_*i*_〉, *y*_*i*_(*t*) − 〈*y*_*i*_〉), the displacement correlation matrix is calculated as *D*_*ab*_ = 〈*u*′_*a*_(*t*)*u*′_*b*_(*t*)〉, where *u*′_*i*_(*t*) = *u*_1_(*t*), *u*_2_(*t*), …, *u*_*N*_(*t*), *v*_1_(*t*), *v*_2_(*t*), …, *v*_*N*_(*t*), *i* = 1, …, *N*, *j* = 1, …, 2*N*, and *N* is the number of particles analyzed. 〈〉 means average over time frames. By diagonalizing *D*_*ab*_, the normal modes are obtained, and the corresponding frequencies are obtained from the eigenvalues *λ*_*a*_, as *ω*_*a*_ = (1/*λ*_*a*_)^1/2^. For the calculation, 898 images, measuring 51.2 *μ*m by 51.2 *μ*m, are taken at 30 frames/s at a height of 25 *μ*m from the coverslip. Figure 4 shows the DOS at polymer concentrations of 0.0 and 1.5 *μ*M. The probability *P*(*ω*) is normalized so that ∫*P*(*ω*)*dω* = 1. The DOS shows a maximum, and the shape is similar to that observed in the previous study^17^. Notable polymer-concentration dependence is not observed in the DOS, indicating that the low-frequency mode is not the cause of the accelerated crystallization.

Finally, the fast relaxation is one of possible causes. In hard-sphere colloidal systems, its correlations with cooperative motion of particles in small clusters^14^ and with the jump of particles to different cages^20^ are discussed. Activation of such particle motions could cause structural rearrangements and accelerate crystallization. Otherwise, the crystallization frequency may correspond to an intrinsic vibrational frequency associated with particle motions in crystal-like structure; when there are clusters showing crystal-like (ordered) structure in a colloidal glass and frequency of applied oscillation matches that of cooperative motion of the clusters, crystalline structure becomes favorable and crystallization is accelerated.

## Conclusions

We found that crystallization in a colloidal glass was accelerated by applying mechanical oscillation at a specific frequency. The frequency increased as the interparticle attractive force increased. Presence of the specific frequency was not explained by the previously proposed model. We considered that there were particle motions associated with crystallization in a colloidal glass, and activation of the motions accelerated the crystallization. Relationships of accelerated crystallization with the slow relaxation and the low-frequency mode were not observed. We speculate that relationships with the fast relaxation and particle motions associated with crystal-like clusters are possible causes, but clear interpretation has not been obtained so far. Our finding reveals a notable relationship between structural change and intrinsic particle motion in a colloidal glass, and this will be analyzed more detail in future works.

## Methods

We use a colloidal suspension consisting of a mixture of water (37.2% by volume) and dimethyl sulfoxide (DMSO) (62.8% by volume) with fluorescein sodium salt (FSS) and silica particles of 1.51-*μ*m diameter with a polydispersity of 3.0%. The solvent nearly matches the refractive index of the particles. FSS concentration is 1.0 *μ*M, and the volume fraction of the colloidal particles is ∼0.025.

**Figure 5:**
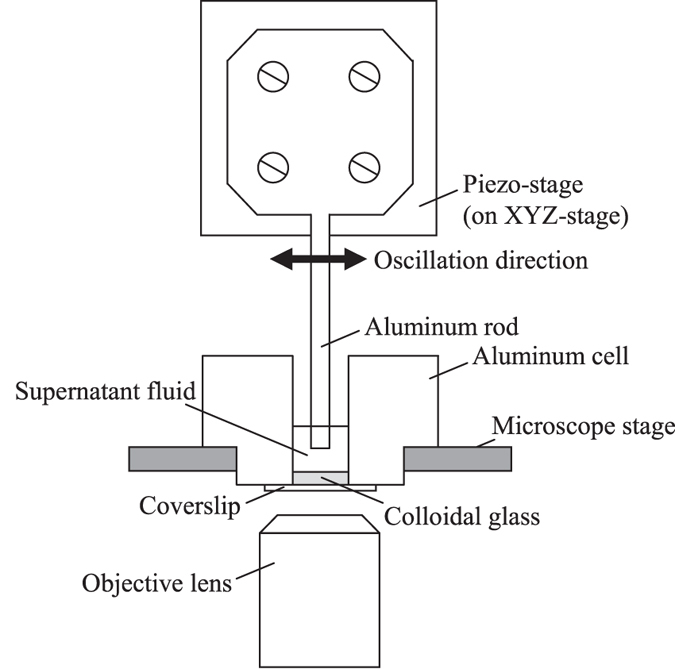
Schematic image of measurement setup.

Figure 5 shows the measurement setup. An aluminium sample cell is used. A through hole of 9-mm diameter is machined in an aluminium block, and a coverslip is attached on a bottom surface of the block. The suspension is poured in the sample cell, and a colloidal glass is formed on the coverslip by the centrifugation with the centrifugal acceleration of about 1850 *g*, where *g* = 9.8 m/s^2^. The height of the colloidal glass is about 70 *μ*m. On the upper surface of the coverslip, silica particles of 5-*μ*m diameter are attached using polymethyl methacrylate (PMMA) to prevent crystallization on the coverslip. Making a rough surface on walls has been used in previous shear experiments^16, 29^. An aluminium square rod with the cross sectional area of 3 × 3 mm^2^ is attached to a piezo stage. The piezo stage is mounted on an XYZ stage, and the rod is inserted into the suspension vertically. The rod is not in contact with the colloidal glass formed on the coverslip. Gap between the top surface of the colloidal glass and the tip of the rod is set to be about 10 *μ*m. Mechanical oscillation is applied by moving the rod in the horizontal direction using the piezo stage. In the most of experiments, structural evolution around a height of 25 *μ*m from the coverslip is evaluated by taking two-dimensional (2D) and three-dimensional (3D) images using a confocal laser microscope. When sample height is changed in Fig. 1(d), 2D images are taken at a height of 20 *μ*m from the coverslip (5 *μ*m lower than other experiments, because of the smaller sample height). The gap between the rod and the colloidal glass is kept to be 10 *μ*m.

The particles’ coordinates are determined by using the code distributed from ref. 30. In a colloidal glass formed by sedimentation, volume-fraction gradation occurs^31^; the volume fraction near the top surface is smaller than that at the bottom. In samples used in the present study, crystallization sometimes occurs near the top surface after a while. However, it is confirmed that crystallization does not occur inside within 2 hours after the sample preparation, which is sufficiently longer than typical experiment time, about half an hour. The volume fraction of particles around at 25-*μ*m height measured by counting the number of particles for a representative sample is 0.59.

Near the top surface of the present samples, crystallization sometimes occurs without applying the mechanical oscillation. One may consider that we are observing the growth of the crystallized regions, not the crystallization inside a colloidal glass. However, as seen in a cross-sectional image taken after 60-s oscillation at 75 Hz (Fig. 1(e)), crystallized regions we observe are not connected with those near the top surface, indicating observed crystallization is not the growth of crystallized regions near the top surface.

## Electronic supplementary material

Supplementary Information
