# Supplementary material for: Accelerated crystallization of colloidal glass by mechanical oscillation
Source: Sci Rep. 2017 May 2;7:1369. doi: 10.1038/s41598-017-01484-y (PMC5430959; doi:10.1038/s41598-017-01484-y)
Supplement: Supplementary file 1 — Supplementary Information [file 41598_2017_1484_MOESM1_ESM.pdf]

Supplementary information

# Accelerated crystallization of colloidal glass by mechanical oscillation

N. Nakamura*, K. Inayama, T. Okuno, H. Ogi, and M. Hirao

Graduate School of Engineering Science, Osaka University, Toyonaka, Osaka 560-8531, Japan

* nobutomo@me.es.osaka-u.ac.jp

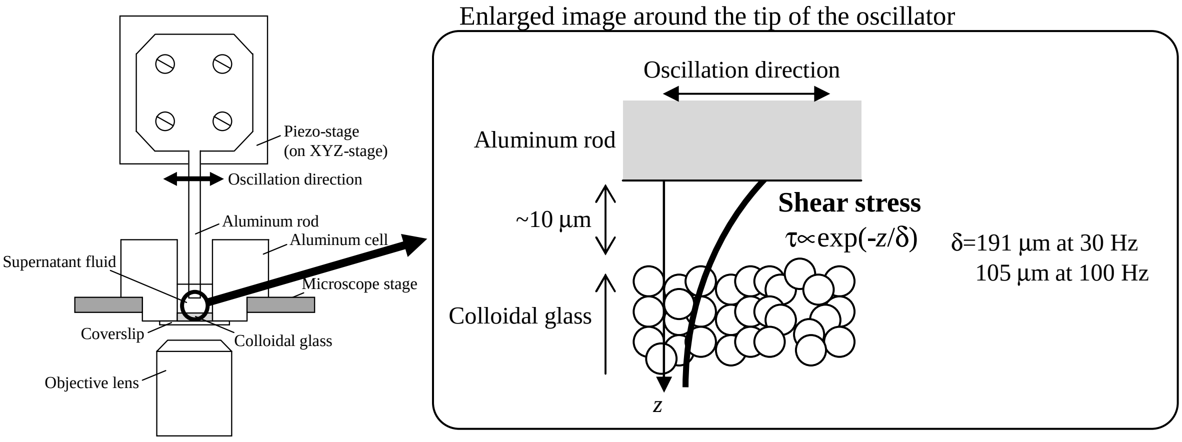


**Supplementary Fig. S1** The measurement setup and the distribution of shear stress applied by the oscillator. The decay length $\delta=(\eta_L/\pi f\rho_L)^{1/2}$ is calculated using the viscosity, $\eta_L$=3.64 mPa· s, and the mass density, $\rho_L$=1059 kg/m$^3$, of the mixture of the water and DMSO; δ=191 and 105 µm at 30 and 100 Hz, respectively. The viscosity was deduced from the reported viscosity of a DMSO-water system [Ref. S1].


**Ref. S1**. Lebel, R. G., & Goring, D. A. I., Density, viscosity, refractive index, and hygroscopicity of mixtures of water and dimethyl sulfoxide, *J. Chem. Eng. Data* **7**, 100 (1962).

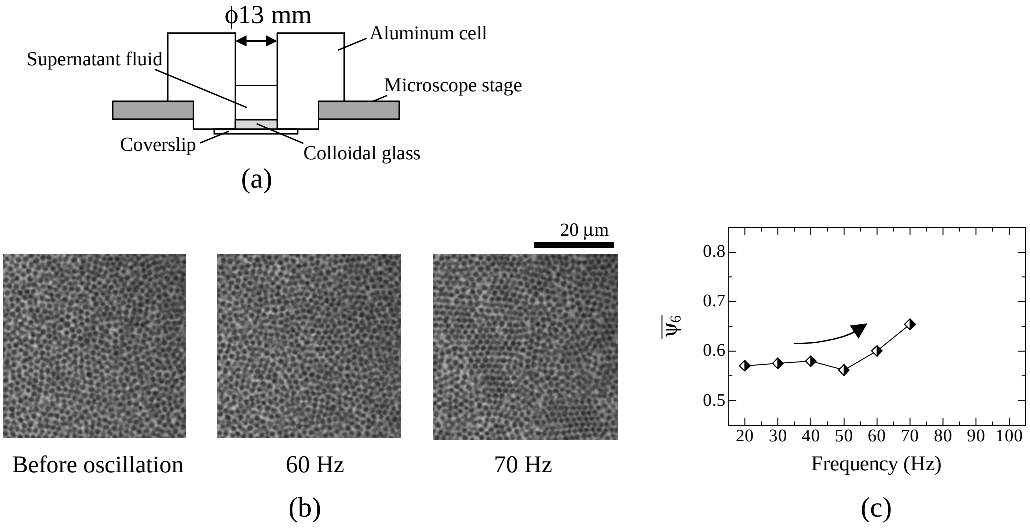


**Supplementary Fig. S2** Experimental result obtained by using the sample cell with the through hole of 13-mm diameter. (a) Schematic of the sample cell, (b) microscopy images taken before oscillation, after 60-Hz oscillation, and 70-Hz oscillation, and (c) the corresponding evolution of the averaged bond orientational order parameter.
